# Suspicious Segmental Pulmonary Artery Aneurysm With Nontuberculous Mycobacterial Infection: A Report of a Rare Case

**DOI:** 10.7759/cureus.109876

**Published:** 2026-05-29

**Authors:** Igal Gorbut, Sheinnera Nayze Gerongay, Priyanka Bhatt, Benjamin Ihde, Robert Subbiondo

**Affiliations:** 1 Internal Medicine, HCA Florida Blake Hospital, Bradenton, USA; 2 Cardiology, Blake Medical Center, University of South Florida (USF), Morsani College of Medicine, Bradenton, USA

**Keywords:** acute hemoptysis, cardiology research, infectious disease medicine, mycobacterial infections, mycobacterium abscessus complex, pulmonary aneurysm

## Abstract

Nontuberculous mycobacteria (NTM) are well-established pulmonary pathogens across all demographics; conversely, pulmonary artery aneurysms (PAAs) represent rare and potentially life-threatening vascular complications. The pathophysiological association between chronic NTM infection and the development of PAAs remains largely underexplored.

A 69-year-old man with a history of untreated NTM infection presented to our facility with a chief complaint of hemoptysis. Initial diagnostic imaging suggested the presence of a PAA in a branch of the pulmonary artery. Consequently, a multidisciplinary consultation ensued to determine the true structural classification of the lesion, weighing a PAA against an acquired, infection-mediated pseudoaneurysm. Subsequent bronchoscopy and culture analysis of the bronchial lavage fluid grew *Mycobacterium abscessus* complex. The patient was managed with intravenous antibiotics and supportive respiratory therapies. Following multidisciplinary consultations, the cardiology service concluded that the PAA was an incidental finding likely secondary to an infectious etiology. The patient was deemed a nonsurgical candidate and was discharged with plans for close outpatient follow-up with cardiology, pulmonology, and infectious disease specialties.

This case details a rare clinical intersection: an incidental PAA discovered during the workup of an active, untreated *M. abscessus* complex infection. While the patient was managed conservatively without surgical intervention, the concurrence of these pathologies underscores a critical diagnostic consideration. Due to the scarcity of documented literature on NTM-associated vascular complications, this presentation highlights the importance of recognizing chronic atypical mycobacterial infections as potential drivers of adjacent vascular wall degradation. Further research is warranted to elucidate the mechanisms by which NTM-mediated inflammation induces vascular wall injury, potentially predisposing patients to aneurysm formation.

## Introduction

Nontuberculous mycobacteria (NTM) comprise over 190 species capable of causing pulmonary pathology across all age groups [1]. Clinically, these are classified by growth rate, with *Mycobacterium avium* complex, *Mycobacterium kansasii*, and *Mycobacterium xenopi* being the most prevalent slow-growing pathogens, while *Mycobacterium abscessus* predominates among rapidly growing strains [1]. Diagnostic criteria require strict clinical, radiographic, and microbiological correlation. A solitary positive sputum culture is often clinically insignificant, representing environmental contamination; consequently, guidelines mandate at least two separate positive cultures to confirm NTM pulmonary disease [1]. Therapeutic initiation depends heavily on species-specific pathogenicity and virulence. For slow-growing strains or indolent presentations, active surveillance remains the preferred clinical strategy [1].

Pulmonary artery aneurysms (PAAs) represent an exceptionally rare vascular pathology. Autopsy data demonstrated an incidence of merely eight cases across 109,571 postmortem examinations [2]. Unlike systemic arterial aneurysms, PAAs demonstrate no distinct gender predilection and predominantly manifest in younger cohorts [2]. Anatomically, the majority of PAAs compromise the main pulmonary trunk, whereas involvement of the peripheral pulmonary branches remains a minority presentation. The underlying etiology is highly variable, traditionally classified into congenital anomalies (congenital heart disease, connective tissue disorders) or acquired insults. Among infectious drivers, syphilis and tuberculosis (TB) remain historical benchmarks. Notably, localized intraparenchymal PAAs arising secondary to cavitary TB, termed Rasmussen aneurysms, typically manifest within the upper lobes, driven by localized progressive chronic inflammation and subsequent vascular wall degradation [3]. While congenital anomalies account for the majority of presentations, other etiologies include acquired idiopathic degradation, iatrogenic injury, systemic vasculitis, autoimmune diseases, underlying malignancies, and diverse infectious processes [4].

## Case presentation

**Figure 1:**
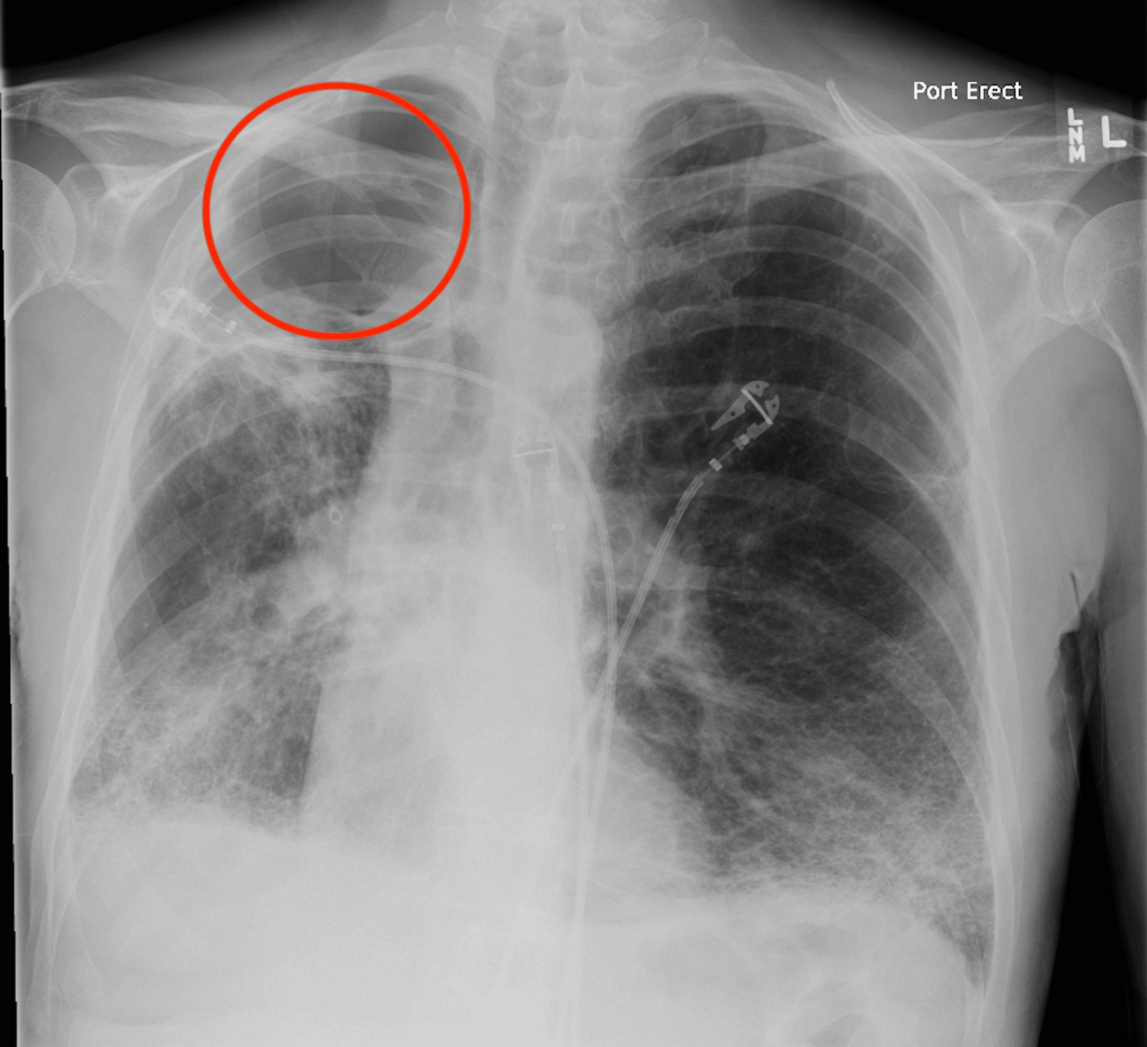
Anterior-posterior chest X-ray taken on admission. Bilateral patchy airspace opacities, right greater than left. Superimposed chronic fibrotic lung changes. Increased lung lucency, likely from emphysema. Circled in red is a large bulla at the right upper lobe.

**Figure 2:**
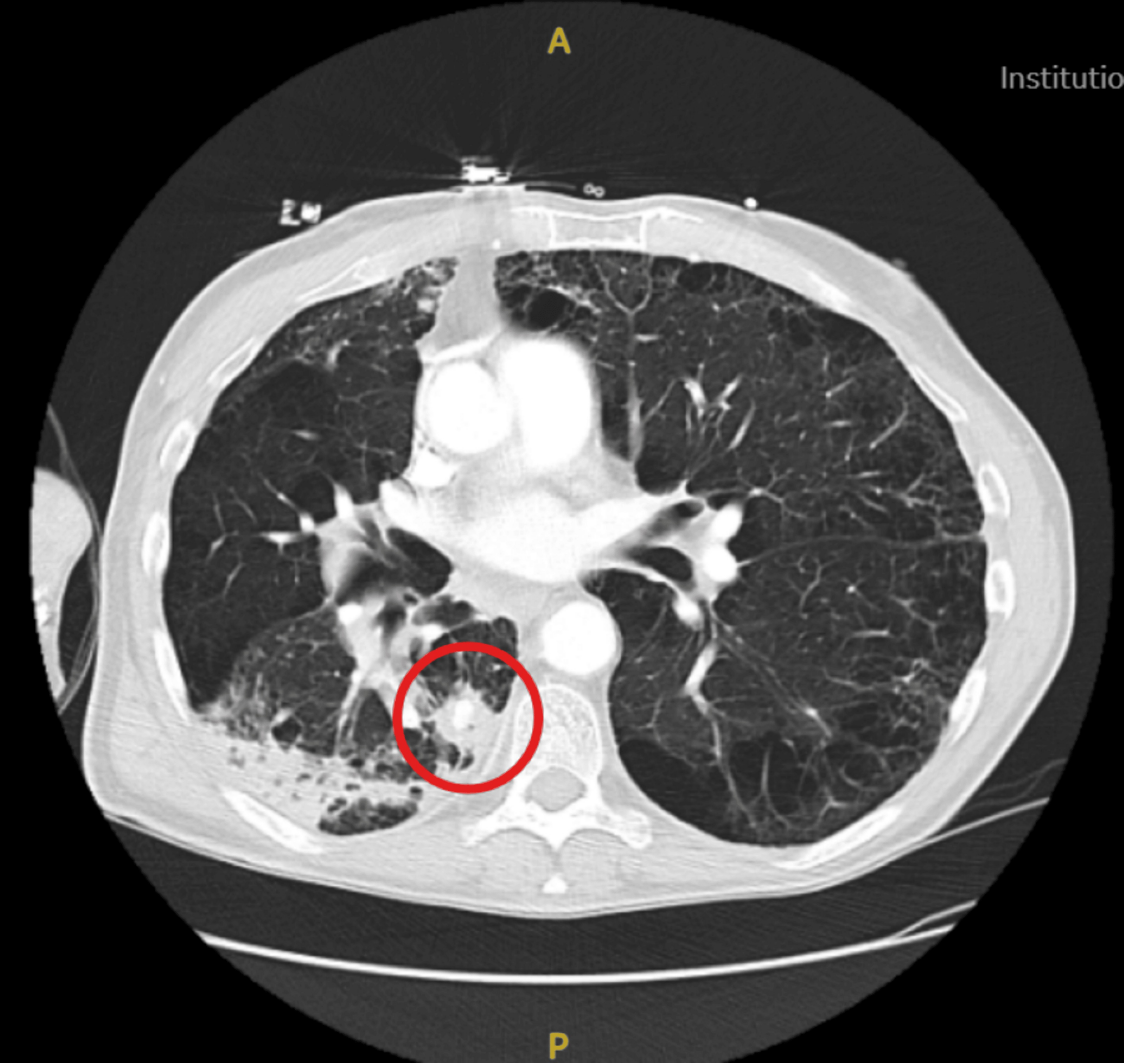
Computer tomography with angiography of the chest, axial section. Circled in red: 2.2 cm nodular opacity in the right lower lobe containing a 10 mm pulmonary artery aneurysm. Severe emphysema and right lower lobe pneumonia. As interpreted by the initial radiology reading.

Our case is regarding a 69-year-old man with a significant history of advanced chronic obstructive pulmonary disease (COPD), severe bullous emphysema, and untreated pulmonary NTM disease, who was transferred to our facility for the management of acute-onset hemoptysis. The patient reported a 24-hour history of progressive dyspnea preceding the hemoptysis. His social history was notable for a comprehensive smoking history of a half-pack per day since age 13. The patient’s initial anterior-posterior chest X-ray showed bilateral patchy airspace opacities, right greater than left, with superimposed chronic fibrotic lung changes, increased lung lucency, likely from emphysema, and a large bulla at the right upper lobe (Figure 1). Initial diagnostic evaluation at the referring facility via a computed tomography angiography (CTA) of the chest revealed a 2.2 cm nodular opacity within the right lower lobe, which enveloped a 10 mm focal PAA. Additionally, the imaging demonstrated severe, widespread emphysematous changes and concurrent right lower lobe parenchymal consolidation consistent with acute pneumonia (Figure 2).

Upon presentation to the referring facility, initial laboratory evaluation demonstrated a profound leukocytosis with a white blood cell (WBC) count of 28 x 10^3^/µL and a serum lactic acid of 3.8 mmol/L. Following transfer to our institution, initial serum diagnostics revealed persistent leukocytosis of WBC 27.7 x 10^3^/µL, normocytic anemia with a hemoglobin level of 10.5 g/dL, and mild hyponatremia with a sodium level of 130 mmol/L, as summarized in Tables 1, 2. Quantitative arterial blood gas (ABG) analysis was attempted; however, further sampling could not be performed due to patient refusal. Hyponatremia was suspected due to poor oral intake, as the patient stated that he had not had adequate oral intake due to malaise for the prior several days, as well as hemoptysis, versus sepsis-induced syndrome of inappropriate antidiuretic hormone secretion. Serum sodium levels normalized following intravenous fluid resuscitation and the restoration of adequate oral intake. The initial elevation in blood glucose normalized on subsequent basic metabolic panels, suggesting a transient, isolated episode of stress-induced hyperglycemia rather than a persistent metabolic derangement.

**Table 1:** Summary of serum chemistry on admission. H: high, elevated value compared to the reference range; L: low, decreased value compared to the reference range

Parameter	Patient value	Reference range
White blood count	27.7 × 10^3^/µL (H)	4.0-10.5 × 10^3^/µL
Red blood count	3.63 × 10^6^/µL (L)	4.63-6.08 × 10^6^/µL
Hemoglobin	10.5 g/dL (L)	13.7-17.5 g/dL
Hematocrit	30.7% (L)	34.1%-44.9%
Mean corpuscular volume	84.6 fL	79.0-92.2 fL
Platelet count	572 × 10^3^/µL (H)	150-450 × 10^3^/µL

**Table 2:** Summary of serum metabolic panel on admission. H: high, elevated value compared to the reference range; L: low, decreased value compared to the reference range; BUN: blood urea nitrogen; GFR: glomerular filtrate rate; CKD-EPI: Chronic Kidney Disease Epidemiology Collaboration; AST: aspartate aminotransferase; ALT: alanine aminotransferase

Parameter	Patient value	Reference range
Sodium	130 mmol/L (L)	136-145 mmol/L
Potassium	4.2 mmol/L	3.5-5.1 mmol/L
Chloride	103 mmol/L	98-107 mmol/L
Carbon dioxide	20.1 mmol/L (L)	21.0-32.0 mmol/L
BUN	18 mg/dL	7-18 mg/dL
Creatinine	0.8 mg/dL	0.8-1.3 mg/dL
Est GFR (CKD-EPI 2021)	>90	>90.0
Glucose	178 mg/dL (H)	74-106 mg/dL
Lactic acid	1.3 mmol/L	0.4-2.0 mmol/L
Calcium	9.2 mg/dL	8.5-10.1 mg/dL
Magnesium	2.0 mg/dL	1.6-2.4 mg/dL
Total bilirubin	<0.2 mg/dL (L)	0.2-1.0 mg/dL
Direct bilirubin	<0.1 mg/dL	0.0-0.2 mg/dL
AST	20 units/L	15-37 units/L
ALT	12 units/L	12-61 units/L
Alkaline phosphatase	88 units/L	45-117 units/L
Total protein	6.8 g/dL	6.4-8.2 g/dL
Albumin	3.7 g/dL	3.4-5.0 g/dL

In the emergency department, the patient was initiated on empiric broad-spectrum antimicrobial therapy, receiving an initial dose of piperacillin/tazobactam 3.375 g before being transitioned to a therapeutic regimen consisting of ceftriaxone 2 g, vancomycin 1,250 mg, and azithromycin 500 mg. The patient required oxygen supplementation via nasal cannula at 2 L/min to maintain adequate oxygen saturation at rest. Given the clinical presentation and history of atypical mycobacterial disease, an infectious workup was immediately initiated to rule out active *Mycobacterium tuberculosis* infection.

The cardiology service was consulted to evaluate the vascular lesion. Following an assessment of the imaging, the consulting team favored a pseudoaneurysm or focal arterial ectasia over a true congenital aneurysm. This diagnostic ambiguity prompted an extensive interdisciplinary discussion between the pulmonology, cardiology, and radiology services regarding the precise structural nature of the vascular anomaly, specifically whether the lesion represented a true, primary aneurysmal dilatation of the vessel wall or an acquired, highly friable infectious pseudoaneurysm. Although catheter-directed angiography is the gold standard for diagnosis, the procedure was deferred because the patient’s acute respiratory dysfunction rendered them clinically unstable, and retrospective imaging review confirmed a highly complex anatomical localization of the vascular anomaly.

They concluded that the localized pulmonary artery dilatation was secondary to a severe, localized infectious etiology, likely compounded by tissue degradation from the patient's extensive, long-standing smoking history. The leading clinical suspicion among the multidisciplinary teams was that the vascular anomaly developed secondary to the patient’s chronic, untreated NTM pulmonary infection along with COPD.

The pulmonology service performed a diagnostic flexible bronchoscopy, which revealed concurrent oral candidiasis (thrush). Endoscopic visualization of the lower airways demonstrated clotted blood combined with viscid mucous secretions partially occluding the mainstem bronchi bilaterally. Notably, there was no evidence of active, ongoing hemorrhage, endobronchial masses, or mucosal ulceration.

Following the procedure, the patient’s respiratory regimen was optimized with nebulized albuterol 2.5 mg and budesonide 1 mg to manage airflow obstruction and localized airway inflammation. Additionally, topical nystatin oral suspension was initiated for the treatment of the oral candidiasis. The patient was initially transitioned to an empirical regimen of cefepime 2 g and vancomycin 750 mg, as the initial scan showed evidence of a right lower lobe pneumonia. However, this regimen was discontinued following the development of a bilateral maculopapular rash isolated to the upper extremities, which was highly suspicious for a drug-induced hypersensitivity reaction.

Serial sputum and tissue cultures were obtained for acid-fast bacilli (AFB). While two separate AFB smear cultures returned positive, the *M. tuberculosis* polymerase chain reaction (PCR) assay was negative. A concurrent serum QuantiFERON-TB Gold assay yielded an indeterminate result.

Microbiological analysis of the bronchial lavage fluid confirmed active growth of *M. abscessus* complex. Final treatment initiation was deferred pending the results of drug-susceptibility testing (DST) to ensure a highly targeted, species-specific antimicrobial regimen. To monitor disease burden and track potential resistance patterns, repeat mycobacterial cultures were obtained. The patient is scheduled for close outpatient follow-up to review the final susceptibility profiles upon organism maturation, at which time definitive tailored therapy will be initiated. Concurrently, upon review of the definitive microbiological data, the infectious disease service discontinued all remaining broad-spectrum systemic antibiotics in the setting of no growth on acute bacterial cultures, shifting focus toward an eventual targeted antimycobacterial management once results of the DST become available.

At the time of discharge, the patient demonstrated significant clinical improvement. His acute dyspnea had markedly ameliorated, the hemoptysis had completely resolved, and he maintained adequate oxygenation on room air, no longer requiring supplemental oxygen at rest. The patient was safely discharged with a structured multidisciplinary outpatient follow-up plan. He is scheduled to be evaluated by both the infectious disease and pulmonology services within three to four weeks. This timeline coincides with the anticipated maturation of the repeat bronchoscopy cultures, allowing the teams to review the final drug-susceptibility profiles and establish a definitive, targeted therapeutic regimen.

## Discussion

PAAs are an exceedingly rare vascular pathology, with historical autopsy data estimating an incidence of approximately one in 14,000 cases [1]. While congenital cardiovascular anomalies account for more than half of all reported presentations, the remaining etiologies comprise a heterogeneous group of acquired insults. These include idiopathic degradation, iatrogenic injury, systemic vasculitis, autoimmune diseases, underlying malignancies, and diverse infectious processes [4]. This discussion focuses specifically on infectious pathogens as primary drivers of PAA pathogenesis.

A retrospective review of the literature suggests that infectious PAAs characteristically manifest in patients ranging from 38.5 to 66 years of age [4]. The predominant initial clinical triad consists of hemoptysis, fever, and a productive cough. Morphologically, infectious aneurysms vary significantly from their congenital counterparts as they have an average documented diameter of 32.7 mm and demonstrate a distinct predilection for the distal pulmonary arterial branches rather than the central trunk [4]. Given the severe risk of catastrophic rupture, definitive management typically necessitates aggressive intervention, including transcatheter embolization, endovascular plug deployment, or surgical lobectomy [5].

Historically, the most common bacterial etiologies associated with infectious pulmonary artery dilatation are *Treponema pallidum* (syphilis) and *M. tuberculosis* (TB). In tertiary syphilis, aneurysmal degeneration characteristically compromises the central, large pulmonary arteries via chronic endarteritis obliterans involving the vasa vasorum. Conversely, advanced, cavitary TB predisposes patients to localized intraparenchymal pulmonary pseudoaneurysms, classically recognized as Rasmussen aneurysms [4]. Typically localizing to the upper lobes during reactivated disease, Rasmussen aneurysms develop as a direct consequence of progressive parenchymal inflammation that erodes the adjacent peripheral pulmonary arterial branches [4].

Based on the radiographic findings and acute clinical presentation of our patient, the vascular anomaly was highly suggestive of a pseudoaneurysm rather than a true congenital aneurysm. This distinction is critically important; pseudoaneurysms lack a true structural vessel wall, rendering them highly friable and inherently prone to catastrophic, life-threatening hemorrhage-consistent with the patient's presenting symptom of acute hemoptysis [5]. The presumptive clinical diagnosis of a low-diameter pseudoaneurysm, combined with its relatively stable dimensions, guided the cardiology and multidisciplinary teams to opt against acute surgical or endovascular intervention. Instead, a conservative, medically driven strategy was pursued, prioritizing targeted antimicrobial stabilization over high-risk operative management.

Catheter-directed pulmonary angiography remains the definitive gold standard for the diagnosis of pulmonary arterial aneurysms and pseudoaneurysms. This invasive modality provides precise visualization regarding the exact extent of vascular involvement, allows for the concurrent hemodynamic assessment of right-sided cardiac pressures, and offers a dual pathway for immediate, simultaneous endovascular therapeutic interventions [5]. In this case, following interdisciplinary evaluation by the cardiology and endovascular teams, the patient was deemed an unsuitable candidate for acute transcatheter intervention or endovascular repair due to the challenging and complex anatomical localization of the pseudoaneurysm.

Although a direct, definitive causal relationship between the *M. abscessus* complex infection and the development of this vascular anomaly could not be established objectively, clinical management proceeded under the strong presumptive diagnosis that the underlying mycobacterial pathology was the primary driving etiology. Management was aligned with proposed therapeutic algorithms for infection-mediated pulmonary artery anomalies, which mandate aggressive treatment of the underlying precipitating etiology as the primary stabilizing measure [2]. Throughout the inpatient stay, clinical efforts focused on supporting the patient through acute hypoxic respiratory failure and managing the concurrent right lower lobe pneumonia. Under this conservative medical regimen, the patient’s hemoptysis completely resolved, and his pulmonary function returned to baseline, precluding the need for supplemental oxygen at the time of discharge. Definitive management of the chronic *M. abscessus* complex infection was deferred to the outpatient setting, with a structured plan for close follow-up with infectious disease specialists to initiate targeted antimycobacterial therapy upon the final maturation of repeat susceptibility profiles.

Recognized pleuropulmonary complications of NTM infections include progressive pleural disease, spontaneous pneumothorax, empyema, and bronchopleural fistulas [6]. However, unlike the well-documented vascular invasion seen in tertiary syphilis and *M. tuberculosis*, where direct transmural inflammation destroys the structural integrity of the vasa vasorum or vessel walls [4], a definitive mechanism directly linking NTM infections to the pathogenesis of PAAs remains unestablished. Although causality cannot be definitively derived from a single case, this presentation remains highly valuable due to its unique conservative management strategy and the extreme rarity of both pathologies presenting concurrently. Given the extreme scarcity of documented vascular anomalies secondary to atypical mycobacteria, and particularly *M. abscessus* complex, further clinicopathological research may be warranted.

Given the necessity of a prolonged and complex antimicrobial regimen for the management of NTM infections, inpatient care explicitly prioritized comprehensive discharge counseling and proactive health advocacy. Because the patient had an established history of NTM infection prior to this admission, the multidisciplinary team focused extensively on mitigating potential barriers to medication adherence, specifically addressing socioeconomic constraints. Establishing a robust therapeutic rapport with the patient was paramount to ensuring long-term outpatient follow-up. Identifying these socioeconomic determinants of health early allowed for the integration of social work and pharmacy assistance resources, optimizing the patient's ability to safely acquire and sustain the multidrug therapy required to treat his underlying disease.

## Conclusions

While definitive causal relationships cannot be established from an isolated clinical observation, this case highlights an exceedingly rare and instructive intersection of *M. abscessus* complex infection and localized pulmonary artery degeneration. Clinical management of such complex vascular anomalies must carefully weigh the virulence and growth kinetics of the specific NTM species against the procedural risks of intervention. When severe respiratory dysfunction and challenging anatomical localizations preclude invasive surgical or endovascular repair, a medically driven strategy prioritizing targeted antimicrobial stabilization remains a viable therapeutic pathway. Ultimately, this presentation emphasizes the importance of early diagnostic vigilance, interdisciplinary collaboration, and the proactive mitigation of socioeconomic barriers to ensure adherence to the prolonged multidrug regimens required to treat the underlying infectious angiopathy.
